# A Novel PZT Pump with Built-in Compliant Structures

**DOI:** 10.3390/s19061301

**Published:** 2019-03-15

**Authors:** Qibo Bao, Jianhui Zhang, Ming Tang, Zhi Huang, Liyi Lai, Jun Huang, Chuanyu Wu

**Affiliations:** 1 College of Mechanical and Electrical Engineering, Guangzhou University, Guangzhou 510006, China; 2111707005@e.gzhu.edu.cn (Q.B.); 2111707033@e.gzhu.edu.cn (M.T.); 2111807019@e.gzhu.edu.cn (Z.H.); 2111807002@e.gzhu.edu.cn (L.L.); 2 National Research Center of Pumps, Jiangsu University, Zhenjiang 212013, China; huangjun@ujs.edu.cn; 3 Faculty of Mechanical Engineering & Automation, Zhejiang Sci-Tech University, Hangzhou 310018, China; cywu@zstu.edu.cn

**Keywords:** PZT pump, valved pump, valveless pump, Compliant structures, flow rate

## Abstract

Different to the traditionally defined valved piezoelectric (PZT) pump and valveless PZT pump, two groups of PZT pumps with built-in compliant structures—with distances between the free ends of 0.2 mm (Group A) and 0 mm (Group B)—were designed, fabricated, and experimentally tested. This type of pump mainly contains a chamber 12 mm in diameter and 1.1 mm in height, a PZT vibrator, and two pairs of compliant structures arranged on the flowing channel. The flow-resistance differences between these two groups of PZT pumps were theoretically and experimentally verified. The relationships between the amplitude, applied voltage and frequency of the PZT vibrators were obtained experimentally, with results illustrating that the amplitude linearly and positively correlates with the voltage, while nonlinearly and negatively correlating to the frequency. The flow rate performance of these two groups was experimentally tested from 110–160 Vpp and 10–130 Hz. Results showed that the flow rate positively correlates to the voltage, and the optimum flow rate frequency centers around 90 Hz for Group A and 80 Hz for Group B, respectively. The flow rate performances of Group B were further measured from 60–100 Hz and 170–210 Vpp, and obtained optimal flow rates of 3.6 mL/min at 210 Vpp and 80 Hz when ignoring the siphon-caused backward flow rate. As the compliant structures are not prominently limited by the channel’s size, and the pump can be minimized by Micro-electromechanical Systems (MEMS) processing methods, it is a suitable candidate for microfluidic applications like closed-loop cooling systems and drug delivery systems.

## 1. Introduction

Microfluidics, where micropumps are the essential components [1,2,3], has shown ever-increasing possibilities for improving diagnostics and biology research [4]. Thanks to the historical finding of the piezoelectric effect by Pierre and Jacques Curie in 1880 and the verification of the inverse piezoelectric effect in 1881, piezoelectric materials have made tremendous progress in the past two centuries. Lead Zirconate Titanate (PZT), a type of artificial piezoceramic material, has a strong piezoelectric effect. With its dual sensing and actuating capacity and wide bandwidth [5,6,7], the PZT material has many engineering applications, such as in vibration sensing [8,9,10], vibration control [11,12,13], ultrasonic wave generation and sensing [14,15], energy harvesting [16,17,18], and structural health monitoring [19,20,21,22]. In addition, PZT material can be fabricated into different sizes and shapes. PZT is a commonly used solid-state actuator, taking advantage of its strong inverse piezoelectric effect. 

PZTs are commonly used in a micropump [23,24,25]. A PZT pump is one kind of pump that manipulates fluid by mechanical deformation caused by a PZT actuator. Up to now, according to the driving principle, PZT pumps can generally be divided into three types: surface acoustic wave (SAW) pumps, peristaltic pumps, and volumetric pumps. The SAW pump, which uses the traveling wave generated by PZT material to drive the liquid in the flow channel, is generally suitable for cases where flow channel sizes are small. Although interdigital transducers (IDTs) [26] are the key components of the SAW pumps, there are also studies on the arrangement of electrodes on PZT tubes to generate traveling waves to deliver fluid [27]. The peristaltic PZT pump utilizes the mechanical deformation of the PZT membrane to cause expansion and contraction of the pump chamber [28,29,30,31,32,33], making the chamber function as a fluidic switch. The peristaltic PZT pump has relatively flexible control methods that enable PZT membranes to actuate in different sequences [34] and to pump the fluid bidirectionally [35]. Generally, the number of pump chambers of the peristaltic PZT pump is not less than three. However, scholars have tactfully designed a peristaltic pump with a single pump chamber [30].

The volumetric PZT pump utilizes the deformation of PZT membrane to cause periodic expansion and compression of the pump-chamber volume; when combined with valve or mechanism which acts as a fluidic diode, one-way flow is possible [2,36,37,38]. The structure, fabrication and controlling method are much simpler than that of the other two types of PZT pumps mentioned above. The research on the volumetric PZT pumps is mainly carried out in two directions: one focuses on the volumetric changing rate by the PZT stack or the leveraging effect [39], and the other one focuses on the flow-resistance mechanism, which is the essence of a volumetric PZT pump. For volumetric PZT pumps, the flow-resistance mechanisms are their ‘valves’ which appear either as visible valves or invisible valves. There are many types of visible valves—such as ball valves, bridge valves, membrane valves, cantilever valves, and wheel valves—which have appeared in the valved PZT pumps [40]. The invisible valves include nozzle/diffuse tubes [41,42], spiral flow tubes by Coriolis force [43], special flow tubes by Coanda effect [44], flow tubes by centrifugal force [45], Tesla flow tubes [46,47], Y-shaped tubes [48,49], block components arranged on the flow channel or on the pump chamber like triangular prisms [50], and asymmetry-slope blocks [51], etc. However, PZT pumps with a valve might be easily fatigued and damaged, have relatively high structural complexity, and can generally obtain higher flow rate and back pressure than valveless PZT pump. The valveless PZT pumps, however, have simpler structures and are easier to be miniaturized [1,52], though generally have a lower flow rate and back pressure.

In order to obtain relatively high flow rates as well as be easily miniaturized, a PZT pump with built-in compliant structures with the ‘valve’ features of both valved and valveless PZT pumps is proposed. Firstly, the designing principle and the pumping feasibility of the pump are illustrated and theoretically verified. Then the prototype and its fabricating process are described. Several experiments were carried out to: verify the differences of the flow-resistance of the pumps, explore the amplitude-voltage-frequency relationship of the vibrators, and to test the flow performance of the pumps. Lastly, the factors which influence the flow rate of the proposed PZT pump were studied and discussed, and future investigations were also discussed.

## 2. Working Principle and Theoretical Analysis

### 2.1. Working Principle

**Figure 1:**
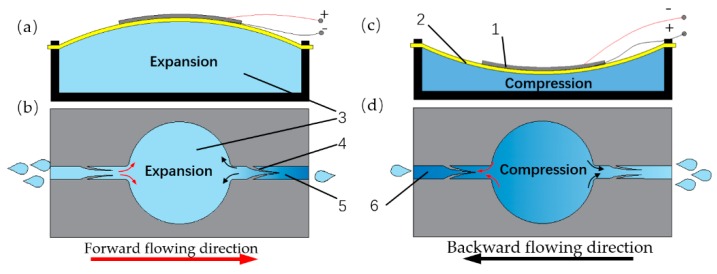
The working principle of the proposed Lead Zirconate Titanate (PZT) pump: (**a**) the pump chamber in suction stroke; (**b**) the inhaling principle during suction stroke; (**c**) the pump chamber in compression stroke; (**d**) the exhausting principle during compression stroke; (1) the piezoelectric ceramic; (2) the brass substrate; (3) the pump chamber; (4) the compliant structure; (5) the outlet channel; (6) the inlet channel.

As shown in Figure 1, the pump is composed of a pump chamber, a PZT vibrator, two flow channels, and two pairs of compliant structures distributed on both sides of the chamber. When the vibrator bulges upward (Figure 1a), the volume of the pump chamber becomes larger, enlarging the gap of the inlet compliant structures while shrinking the gap of the outlet compliant structures, making more fluid pass through the inlet channel than the outlet channel (Figure 1b). Vice versa, when the vibrator recesses downward, the volume of the pump chamber becomes smaller (Figure 1c), enlarging the gap of the outlet compliant structures while shrinking the gap of the inlet compliant structures, making more fluid pass through the outlet channel than the inlet channel (Figure 1d). More fluid flows into the pump chamber from the inlet channel than that from the outlet channel during the suction stroke, and more fluid flows out from the outlet channel than that from the inlet channel during the compression stroke. As long as the vibrator is driven by an alternating excitation signal to cause periodic volume change, the fluid can be continuously delivered from the inlet channel to the outlet channel.

### 2.2. Theoretical Analysis

When the flow state of the fluid is laminar and Newtonian, the relationship between the pressure drop, the flow-resistance, and the flow rate can be described by Poiseuille’s law [53]:

(1) $$\Delta P=\frac{8\mu LQ}{\pi R^{4}}$$

where ΔP is the pressure drop at the two ends of the flow channel, L is the length of the channel, μ is the dynamic viscosity of the fluid, Q is the flow rate of per unit volume, and R is the channel’s radius. Since the cross-section of the flow channel of the proposed PZT pump is rectangular, and since the ratio between the width and the height of the channel is 4:3, the R of Equation (1) can be replaced by the hydraulic diameter $d_{h}$ of the rectangular flow channel:

(2) $$d_{h}=\frac{4A}{p}$$

where A is the flow area when the fluid is flowing through the channel, p is the wet perimeter—defined as the total contacting length of the channel boundary and the fluid. Substituting $d_{h}$ into Equation (1) gives,

(3) $$\Delta P=\frac{128\mu LQ}{\pi d_{h}^{4}}$$

The flow rate of the pump can be calculated [54] by:

(4) $$Q=\Delta Vf\xi_{y}\xi_{v}$$

where ΔV is the volume-change quantity of the pump chamber, f is the driving frequency, $\xi_{y}$ is the flow-resistance along the channel, and $\xi_{v}$ is the flow-resistance of the compliant structures. For the circle PZT vibrator, which covers the pump chamber, the volume-change quantity can be simplified [55] to:

(5) $$\Delta V=\pi \omega_{0}r^{2}$$

where $\omega_{0}$ is the maximum amplitude of the PZT vibrator, r is the radius of the substrate of the PZT vibrator. According to [54], the flowing resistance due to compliant structures can be calculated by:

(6) $$\xi_{v}=\frac{\bar{\xi {\left(\chi \right)}_{+}}-\bar{\xi {\left(\chi \right)}_{-}}}{\bar{\xi {\left(\chi \right)}_{+}}+\bar{\xi {\left(\chi \right)}_{-}}}$$

(7) $$\bar{\xi {\left(\chi \right)}_{+}}=\frac{\int_{\chi \to \infty }^{\;}\xi {\left(\chi \right)}_{+}d\chi }{\chi }$$

(8) $$\bar{\xi {\left(\chi \right)}_{-}}=\frac{\int_{\chi \to \infty }^{\;}\xi {\left(\chi \right)}_{-}d\chi }{\chi }$$

$\bar{\xi {\left(\chi \right)}_{+}}$ is the average flow-resistance when the fluid is flowing through the compliant structures when the gap between the free ends of the compliant structures is enlarged; $\bar{\xi {\left(\chi \right)}_{-}}$ is the average flow-resistance when the fluid is flowing through the compliant structures when the between the free ends of the compliant structures is decreased; χ is the elapsed time.

One of the essential requirements for a volumetric pump like the proposed PZT pump is that $\xi_{v}$ cannot be zero. However, for any pair of compliant structures, it can be satisfied:

(9) $$\bar{\xi {\left(\chi \right)}_{+}}-\bar{\xi {\left(\chi \right)}_{-}}\neq 0$$

## 3. Fabrication

With the enrichment of materials and the diversification of processing methods, for the current PZT pump, the manufacturing materials can be selected from polydimethylsiloxane (PDMS), polyethylene, SU-8, hydrogel, perylenes, among others. The manufacturing methods can be selected from CNC machining, mold forming, 3D printing technology, and MEMS processing methods such as deep reactive ion etching, wet etching, etc. 3D printing is an ideal choice for prototyping, and its ever-increasing printing quality can easily meet the accuracy requirements. The stereolithography apparatus (SLA, SL300, ZRapid Tech, Suzhou, China) with a printing accuracy of 10 μm was used to print the pump body, and the transparent photosensitive resin was used to obtain transparent pump-body structures. However, due to the poor flow of the resin in the micro flow channel, the flow path could not be designed too small. 

**Figure 2:**
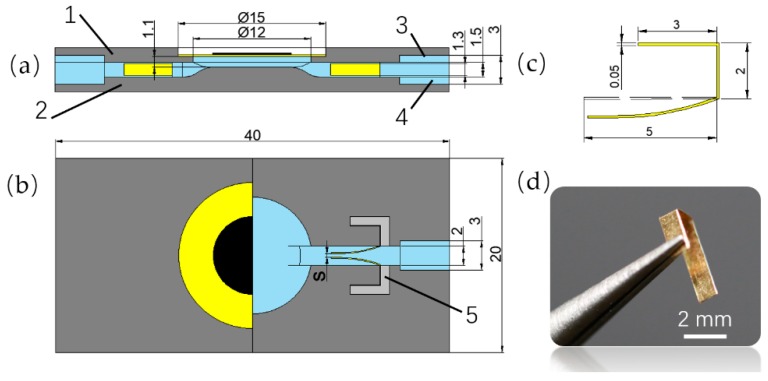
Specific dimensions of the PZT pump: (**a**) the main sectional view of the PZT pump; (**b**) the top view of the PZT pump with a half of its upper part; (**c**) the main dimensions of the compliant structure; (**d**) the fabricated compliant structure; (1) the upper part; (2) the lower part; (3) the groove of the upper part; (4) the groove of the lower part; (5) the groove of the compliant structure.

The pumps were divided into the upper part and the lower part to be separately printed, and its corresponding dimensions are shown in Figure 2. The upper part included a PZT vibrator mount, and two cannula grooves at both ends of it. The lower part included a pump chamber, an inlet channel, an outlet channel, four grooves arrayed on both sides of the pump chamber to mount the compliant structures, and two cannula grooves at both ends of the flowing channels. The overall length, width, and height of the pump were 40 mm, 20 mm, and 4.5 mm, respectively. The diameter and height of the pump chamber were 12 mm and 1.1 mm, respectively. The width and the height of the flow channel are 2 mm and 1.5 mm, respectively. Two square cannula holes with a cross-sectional dimension of 3 mm × 3 mm were formed as the combination of the upper part and the lower part. The compliant structure was made of a brass sheet with a thickness of 0.05 mm, and its dimensions are shown in Figure 2c. The fabricated compliant structure is shown in Figure 2d. And the main dimensions of the brass-based PZT vibrator (KS-152T60A, COSSON, Dongguan, China) are shown in Table 1.

**Figure 3:**
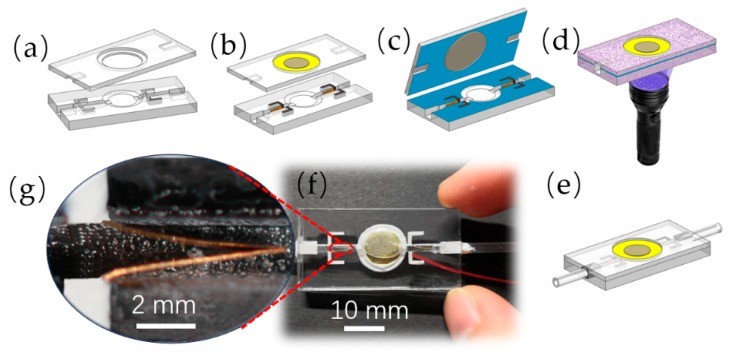
The fabricating process of the PZT pump: (**a**) the upper part and the lower part; (**b**) the attachment of the PZT vibrator and the compliant structures; (**c**) the coating by UV epoxy; (**d**) UV-curing; (**e**) the hoses intubating; (**f**) the fabricated PZT pump; (**g**) a detailed view of the assembled compliant structural pair.

The manufacturing process of the pump is mainly shown in Figure 3. The upper part and the lower part (Figure 3a) were properly cleaned after printing, and the compliant structures were mounted on the mounting grooves by tweezer. The silicone was injected into the interspace in the mounting grooves by a small syringe, and at the same time the vibrator was attached to the vibrator mount by silicone (Figure 3b). After the silicone cured for 3 h, the upper part and the lower part were ready to be bonded together. In order for a fast bonding speed, the upper part and the lower part were bonded by ultraviolet (UV) epoxy. In a dark environment, the UV epoxy was coated on the lower surface of the upper part and the upper surface of the lower part (Figure 3c). Then these two parts were accurately clamped together by several clips and bonded by 30 s of UV light exposure (Figure 3d). Two plug ports (cannula holes, as mentioned before) were formed on both ends of the PZT pump after bonding, into which two PVC hoses were inserted and fixed and sealed with silicone (Figure 3e). The finished PZT pump is shown in Figure 3f and one of its compliant structural pairs is shown in Figure 3g.

In order to separately compare the influence of different initial conditions—defined by the distance S between the free ends of the compliant structures (Figure 2b)—on the performance of the PZT pump, the pumps were divided into Group A and Group B. For Group A, the distance was 0.2 mm; and for Group B, the distance was 0 mm.

## 4. Experimental Setups

There are three main purposes for setting up the experiments: to verify the actual existence of the flow-resistance difference which is the essential requirement of the pumping capability, to obtain the relationships between the amplitude, voltage, and frequency of the vibrator, and to test the pumping performance of the pumps.

The working medium was distilled water which was used after boiling and cooling down to room temperature. The experimental conditions were indoors and no wind, and the room temperature was 17 °C. The peripheral instruments mainly used were a function signal generator (AFG1062, Tektronix, Beaverton, WA, USA), an oscilloscope (DSO-X2004A, Keysight, Santa Rose, CA, USA), a power amplifier (HVP-300D, NJFN, Nanjing, China), and a laser displacement sensor (LK-H020, Keyence, Osaka, Japan).

### 4.1. Measurement of the Flow-Resistance Differences

**Figure 4:**
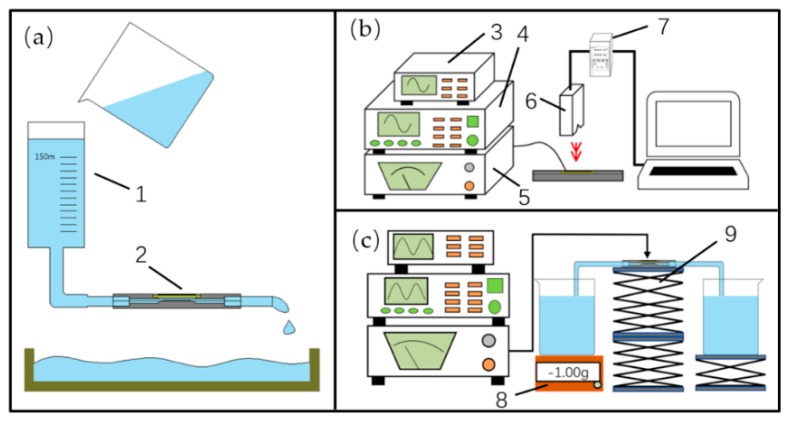
Experimental setups: (**a**) the experiment of flow-resistance difference; (**b**) the amplitude measurement of the PZT vibrator; (**c**) the flow rate measurement; (1) syringe barrel; (2) the PZT pump; (3) oscilloscope; (4) signal generator; (5) power amplifier; (6) laser sensor; (7) the controller; (8) electronic balance; (9) lifting platform.

The flow-resistance difference of the pump can be verified by a simple experiment whose measuring principle is shown in Figure 4a. A 150 mL syringe barrel was hung at a height of 910 mm and connected to the pump by a PVC hose with an inner diameter of 5 mm. Then the water was poured into the syringe barrel to flow through the pump. In order to reduce the formation of bubbles, the deposition of steam or other impurities was cleaned by pouring ethanol (99.5%) into the hose, and the residual ethanol was cleaned by water. After making sure there were no air bubbles in the hose and the pump, the elapsed time for the level of the syringe to drop from 150 mL to 20 mL of was recorded. After changing the direction of flow of the pump, the differences in the recorded times were compared and analyzed.

### 4.2. Measurement of the Amplitude Characteristics

As a rule of thumb, the amplitude of the vibrator increases by increasing the applied voltage. However, the higher the voltage is, the easier the PZT ceramic is damaged due to the strain—causing cracks and failure. The amplitudes of the vibrator fixed on the pump body, under different voltages and frequencies, are unknown. 

The measuring principle of the amplitude-voltage-frequency relationships of the vibrator is shown in Figure 4b. The sinusoidal signal was generated by the signal generator, and amplified by the power amplifier, and then applied to the PZT vibrator under monitoring by the oscilloscope. The laser displacement sensor was aimed at the center of the vibrator, and the controller sampled, stored and transmitted the amplitude information to the computer to perform dot matrix measurements. The amplitude information of each measuring file was quickly and easily obtained by using the chart function of Excel (Excel 2016, Microsoft, Albuquerque, NM, USA).

### 4.3. Measurement of the Flow Rate

The measuring principle of the flow rate performance of the pump is shown in Figure 4c. The pump, which placed on the lifting platform, was connected to two PVC hoses whose ends were placed into two beakers. The left beaker was placed on a balance (RC50001, RONGCHENG, Cixi, China) with a resolution of 0.01 g, and was equal in height to the right beaker. Then the pump was filled with water and the two beakers’ water levels were made to be the same before performing the flow rate test. The electronic balance was used to observe and record the weight loss when the pump delivered water from the left beaker to the right beaker over a certain period of time.

## 5. Results and Discussion

### 5.1. Results of the Flow-Resistance Differences

**Figure 5:**
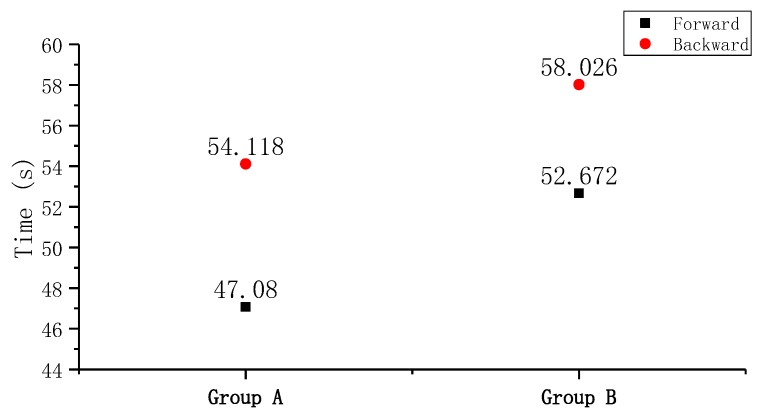
The averaged elapsed time of two groups of PZT pumps in the forward and backward directions.

The elapsed times for forward flow and backward flow of Group A were 47.080 s and 54.118 s; those of Group B were 52.672 s and 58.026 s, respectively. As shown in Figure 5, the forward flow-resistance is smaller than the backward flow-resistance. As the plain view of the pump was axisymmetric except for the compliant structures, the compliant structures constituted the flow-resistance differences between forward and backward direction of flows. Since the gap of the compliant structures of Group A were larger than that of Group B, the elapsed time of Group B was longer than that of Group A, no matter in which direction of flow. However, the difference of elapsed time of Group A (7 s) was larger than that of Group B (5.3 s). In theory, Group A, which has a larger difference in flow-resistance, would obtain a relatively higher flow rate.

### 5.2. Results of the Amplitude Characteristics

**Figure 6:**
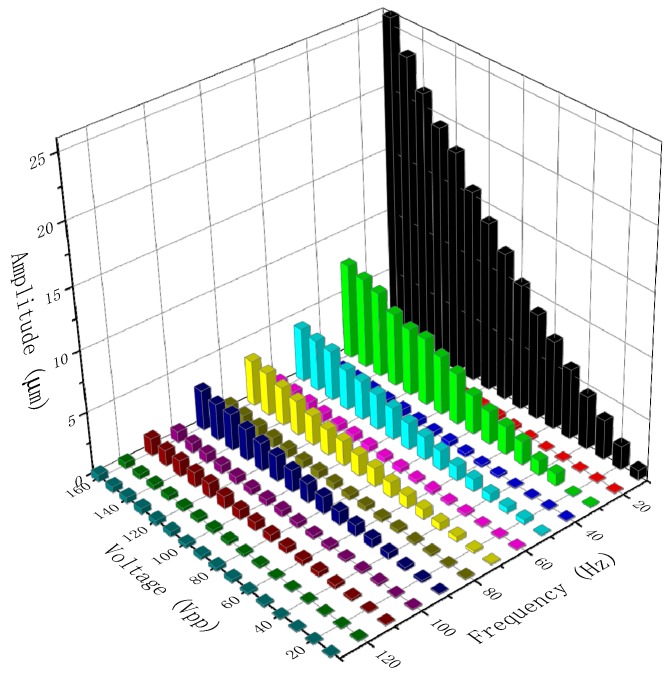
The amplitude-voltage-frequency relationships of the PZT vibrator.

The amplitude-voltage-frequency relationships of the vibrator are shown in Figure 6. At a certain voltage, the amplitude showed a tendency to fluctuate and attenuate with an increase in frequency. This trend is similar to a nonlinear fluctuating attenuating sinusoid, which is mainly determined by the vibrator’s own properties. At a certain frequency, the amplitude of the vibrator increased linearly with increases in applied voltage. It is relatively hard to fit the amplitude-voltage-frequency relationships due to the linear and nonlinear relationships among them. 

**Figure 7:**
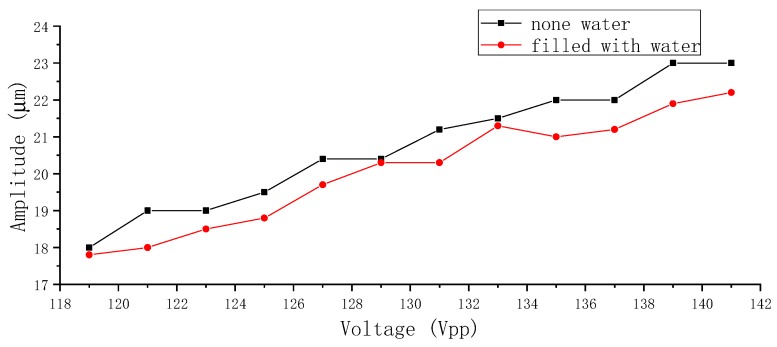
The amplitude of the PZT vibrator under two operating conditions.

Furthermore, it was found that the amplitude’s attenuation of the vibrator had little to do with whether the pump chamber was fulfilled with water. Two comparative experiments to measure the amplitude of the vibrator were carried out at 10 Hz and from 119–141 Vpp with a step of 2 Vpp in two separate conditions: with a pump chamber filled with water and an empty one. The comparative results are shown in Figure 7. The amplitude of the vibrator increased with the increase of the voltage, which is consistent with the measured results in Figure 6. However, the amplitudes of the without-water group were relatively higher than that of the with-water group, though the maximum amplitude difference did not exceed 5%.

### 5.3. Results of the Flow Rate Measurements 

**Figure 8:**
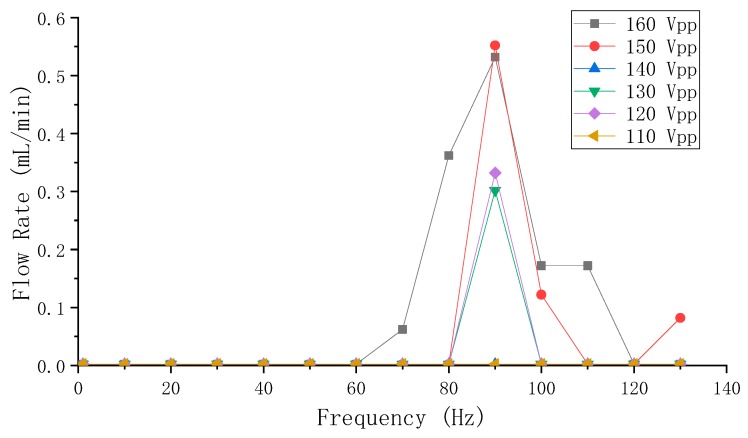
The flow rate measuring results of Group A PZT pumps.

**Figure 9:**
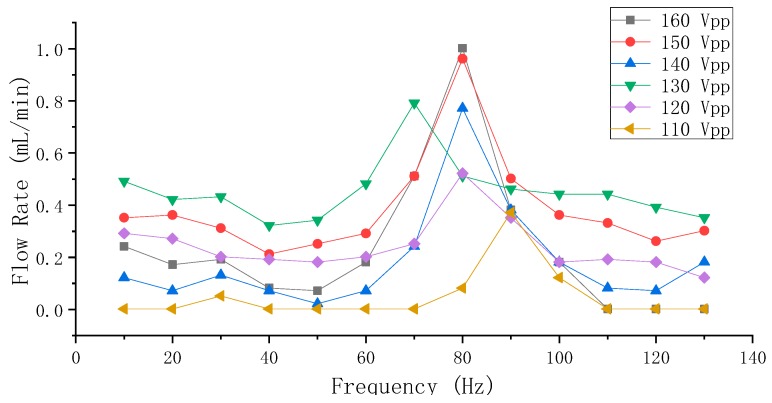
The flow rate measuring results of Group B PZT pumps.

The results of the flow rate measurements of Group A and Group B (from 110–160 Vpp with a step of 10 Vpp and from 10–130 Hz with a step of 10 Hz) are shown in Figure 8 and Figure 9, respectively. 

In Figure 8, it can be observed that the overall flow rate positively correlated to the applied voltage, and the frequency range of the optimum flow rate was centered at 90 Hz. The optimum flow rate was achieved at 160 Vpp and 90 Hz, and was approximately 0.55 mL/min. The flow rate monotonically and sharply decreases on both sides of 90 Hz, and the flow rate is close to zero as the voltage is below 110 Vpp or the frequency is below 70 Hz or above 120 Hz. 

In Figure 9, it can be observed that the influence of frequency on the flow rate of Group B is less obvious than that of Group A, and that the overall flow rate also positively correlates to the applied voltage. However, the frequency range for a high flow rate in Group B mainly occurred around 80 Hz, and the decrease of the flow rate is fluctuant on both sides of 80 Hz. This fluctuant trend could correspond to the amplitude characteristic of the vibrator in Figure 6. The maximum flow rate was achieved at 160 Vpp and 80 Hz, and was 1 mL/min. 

**Figure 10:**
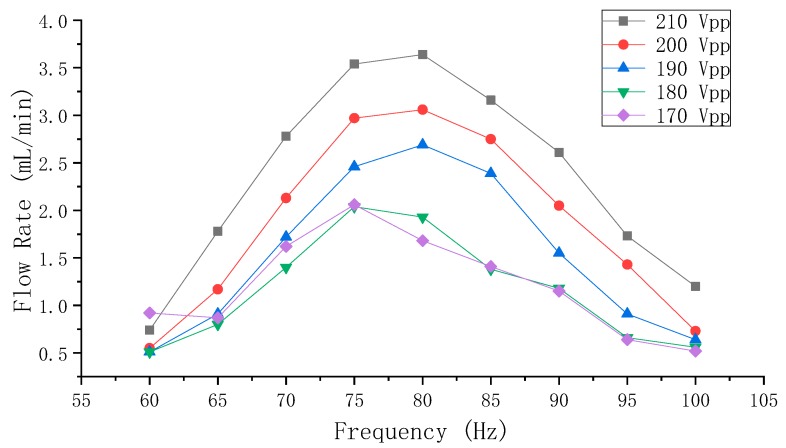
The flow rate results of the Group B PZT pump at the high frequency range.

Since the flow rate increased with the increase of applied voltage and since Group B had better flow rate performance, further experiments were carried out to test the flow rate of Group B from 170–210 Vpp with a step of 10 Vpp and from 60–100 Hz with a step of 5 Hz, and the results are shown in Figure 10. It could be observed that the flow rate still positively correlated to the applied voltage, however, the fluctuant characteristic of the flow rate that was shown in Figure 9 was not obvious when above 180 Vpp. The maximum flow rate, which was limited by the applied voltage, was 3.6 mL/min at 210 Vpp and 80 Hz. Unfortunately, a significant beep would sound when the voltage was above 220 Vpp even if the frequency was maintained from 60–120 Hz, and the PZT ceramic on the vibrator would be highly susceptible to crack damage due to large strain when the voltage reached above 300 Vpp.

Group A should have larger flow rates because of the larger flow-resistance difference compared to Group B. However, the flow rate performance of group B is better, which could be rationalized by several aspects. On the one hand, the siphon-caused backward flow rate of Group A was higher than that of Group B, due to the distance S of Group A being larger than that of Group B. On the one hand, the flow-resistance verifying experiments could only explain that there is a flow-resistance difference between forward and backward pumping directions of the pumps. On the other hand, the free ends of the compliant structure of Group B could attach to each other tighter than that of Group A, efficiently blocking the fluid that flowed out of the chamber to the inlet channel and the fluid that flowed into the chamber from the outlet channel.

As illustrated by Equation (4), the flow rate is proportional to the volume-change quantity and the operating frequency. However, there is a contradiction between the volume-change quantity and the frequency of the vibrator when the voltage is constant: the high frequency makes the volume-change quantity small. Vice versa, the low frequency makes the volume-change quantity large. The increasing frequency influences the volume-change quantity in a fluctuant attenuating way, as shown by the relation of the frequency and the amplitude in Figure 6. However, according to the flow rate results in Figure 8 and Figure 9, it can be known that low (below 70 Hz) or high (above 100 Hz) frequency is not conducive to achieve high flow rates for the pump. The optimum flow rate frequency, which could be considered as the compromised frequency of the system to generate optimum flow rate, is 90 Hz for Group A, and 80 Hz for Group B. Once the distance S changed, the so-called compromise frequency, which is unable to be acquired from a single PZT vibrator by the eigenfrequency analysis, would change accordingly. According to Figure 8, Figure 9 and Figure 10, it can be learnt that the applied voltage does not change the frequency range of the optimum flow rate, and that the applied voltage is independent of the frequency. Higher flow rate could be achieved by increasing the volume-change quantity, as long as the compromised frequency is kept.

**Figure 11:**
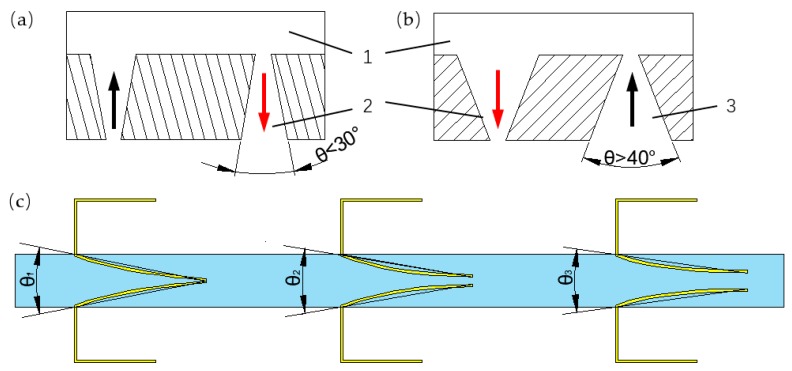
The cone angle of PZT pump: (**a**) the pumping direction when the cone angle was below 30°; (**b**) the pumping direction when the cone angle was above 40°; (**c**) the flare angle change of the compliant structures; (1) the chamber; (2) the outlet; (3) the inlet.

The pumping direction of the valveless pump with cone-shaped tubes can be judged by the cone angle [52]. When the angle was less than 30°, the outlet was the diffuser as shown in Figure 11a; when the angle was more than 40°, the outlet was the nozzle as shown in Figure 11b. It was hard to match the compliant structures pair to the nozzle or diffuser, due to the flare angle of the compliant structures changed by the ever-changing chamber pressure. The flare angle of the compliant structure of the proposed PZT pump was less than 30° and shrunk as the gap of the free ends of the compliant structures enlarged, as shown in Figure 11c. 

### 5.4. Research on the Siphon- Caused Flow Rate

**Figure 12:**
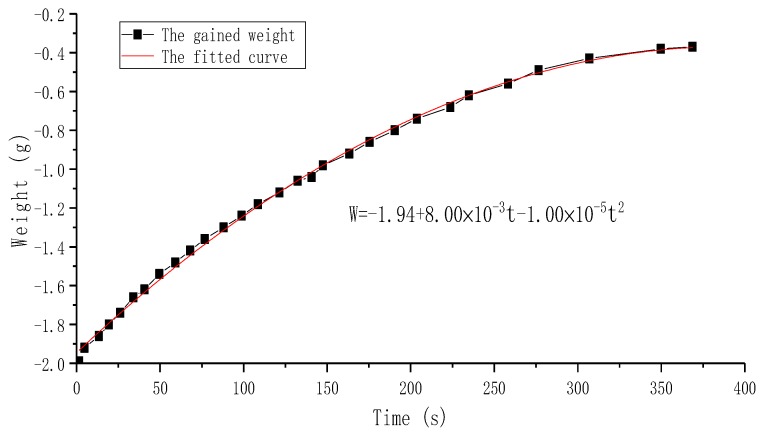
The relationship of siphon-caused gained weight versus time.

The siphon-caused backward flow rate was studied experimentally, and the experimental schematic is shown in Figure 4c. After pumping 2 mL of water to the right beaker (the balance displayed a value of −2.00 g) by the Group B PZT pump, the elapsed time was recorded once the balance gained weight, and the results are shown in Figure 12. A polynomial fitting curve by Origin Lab (OriginPro 2017c, OriginLab, Northampton, MA, USA) was applied to this time-weight relationship, as shown in Figure 12. It is apparent that the growth rate of the curve became slow as the time went by, since the difference in liquid level of two beakers on both sides of the pump gradually decreased. According to the function of the fitted curve, the siphon-caused backward flow rates were calculated to be 0.48 mL/min at 0 s, 0.4 mL/min at 60 s, 0.3 mL/min at 150 s, and 0.12 mL/min at 300 s. Therefore, the siphon-caused backward flow rate of the proposed PZT pump could not be ignored, and could be reasonably compensated to flow rate results in Figure 9 and Figure 10. Considering the siphon-caused backward flow rate would change continuously during forward flow rate measurement, the fitted curve could also be divided into several straight lines whose slopes could be approximately equal to the backward flow rate. Due to the flow-resistance and capillary force of the pump and peripheral piping system, in addition to the low sensitivity of the balance when continuously gaining weight, it was difficult to observe the value change of the balance when the reading was −0.40 g. 

The proposed PZT pump did not have excellent back-pressure performance, as verified by the siphon-caused backward flow rate experiment, partially due to the interspaces between the compliant structures and the flowing channels, and partially due to a lack of sufficient power because the pump chamber was single and small. Furthermore, the back-pressure performance depended on many factors such as the number of the pump chambers and valves [56,57,58], the volume-change quantity of the pump chamber [59], and the resistant properties of the valved mechanisms [60], etc. 

### 5.5. Future Investigations

The research on the PZT pump with built-in compliant structures has just begun, and there are still many problems needed to be addressed. The influence of the dimension and shape of the compliant structures on the flow rate performance, and the limitation of the channel’s size, are unknown. Also, it was found that, in a group besides Group A and Group B, the pumping direction was backward at low frequency and low voltage and was forward at high frequency and high voltage, bidirectional pumping was possible. Since the size of the pump chamber and overall structures were relatively small, and since the compliant structures can be “built” in small-size channels by MEMS methods like deep reactive ion etching, we assume that this type of pump can be a suitable candidate for microfluidics likes closed-loop cooling system and drug delivery system.

## 6. Conclusions

A novel PZT pump, which mainly consists of a pump chamber and a PZT vibrator and two pairs of compliant structures arrayed on the channel, was designed, fabricated, and tested in this paper. The pumps were grouped into Group A and Group B by the distance of the free ends of the compliant structures is 0 mm and 0.2 mm, respectively. The flow-resistance differences between these two groups were verified, and the amplitude-voltage-frequency relationships of the vibrator were tested experimentally to help to explain the flow rate results of Group A and Group B. The flow rate and the vibrator’s amplitude positively related to the applied voltage; however, the vibrator’s amplitude negatively and nonlinearly related to the applied frequency. The compromised frequency for the optimum flow rate, which is resultant of the volume-change quantity of pump chamber and the vibration of the compliant structure, was 90 Hz for Group A and 80 Hz for Group B. The compromised frequency would change by changing the distance mentioned above. Further flow rate tests for Group B had better flow rate performance than Group A, where the achieved maximum flow rate was 3.6 mL/min at 210 Vpp and 80 Hz. The maximum flow rate was limited by the volume-change quantity of the pump chamber, due to the limited applied voltage and the fact that the amplitude of the vibrator was extremely small at the compromised frequency. The influence of the siphon-caused backward flow rate on the forward pumping flow rate results were evaluated, and it was found that the siphon-caused flow rate was high due to the interspace between the compliant structures and the channels, illustrating that the pump would not achieve high back pressures. The compliant structures are not prominently limited by the channel’s size, and the pump can be minimized by MEMS processing methods, making it a suitable candidate for microfluidics applications like closed-loop cooling systems and drug delivery systems.

## Figures and Tables

**Table 1:** The parameters of the PZT vibrator.

Parameter	Diameter of Brass Substrate	Thickness of Brass Substrate	Diameter of PZT Ceramic	Thickness of PZT Ceramic
Value	15 mm	0.1 mm	11 mm	0.1 mm
